# Cryptophytes as potential source of natural antimicrobials for food preservation

**DOI:** 10.3389/fmicb.2024.1462696

**Published:** 2024-09-25

**Authors:** Maryam Abidizadegan, Elina Peltomaa, Polina Ilina, Päivi Tammela, Jaanika Blomster

**Affiliations:** ^1^Ecosystem and Environmental Research Program, Faculty of Biological and Environmental Sciences, University of Helsinki, Helsinki, Finland; ^2^Department of Forest Sciences, University of Helsinki, Helsinki, Finland; ^3^Drug Research Program, Division of Pharmaceutical Biosciences, Faculty of Pharmacy, University of Helsinki, Helsinki, Finland

**Keywords:** cryptophytes, exopolysaccharides, phenolic compounds, antimicrobial activity, food-borne pathogens

## Abstract

Cryptophytes are a promising source of bioactive compounds that have not been fully explored. This research investigated the antimicrobial activity of total phenolic compounds (TPC) and exopolysaccharides (EPS) extracted from several cryptophytes against a range of harmful foodborne bacteria and fungi. To measure the minimum inhibitory concentration (MIC) value, the broth microdilution method was used. In the antibacterial evaluation of TPC, the MIC ranged between 31.25 and 500 μg/mL, while for the antifungal activity test, it varied from 31.25 to 125 μg/mL. In the antibacterial activity test of EPS, the MIC values ranged from 125 to 1,000 μg/mL, whereas in the antifungal susceptibility test, it ranged between 62.5 and 1,000 μg/mL. The most resistant pathogen against TPC was *Escherichia coli*, while *Campylobacter jejuni* was the most susceptible. In the case of EPS, the most resistant pathogen was *Salmonella* Typhimurium, while *Aspergillus versicolor* exhibited the highest susceptibility. Overall, in terms of antimicrobial activity, TPC was more effective than EPS. Finally, the tolerance level (TL) for TPC and EPS was ≤4 in all tested samples, indicating their bactericidal/fungicidal mechanism of action. In conclusion, TPC and EPS isolated from cryptophytes demonstrated remarkable antimicrobial properties and ability to fully eradicate pathogens, and could be considered as natural preservatives in the food industry.

## 1 Introduction

In recent years, the food industry has faced the challenge of improving food production in sustainable ways. To enhance quality standards, food industries are seeking innovative solutions to produce safe and natural food products with extended shelf-life (Cabral et al., 2021; Taylor et al., 2019). Synthetic preservatives interact with the cellular components of the body, resulting in a range of food-related disruptions and detrimental toxicological and allergic effects on human health (Carocho et al., 2015; Martins et al., 2017; Pinto et al., 2023; Sambu et al., 2022). Common synthetic preservatives include sodium benzoate, potassium sorbate, sulfur dioxide, and calcium propionate, which have potential health risks such as allergies and sensitivities (Gupta and Yadav, 2021). Additionally, when used on an industrial scale, synthetic chemical compounds result in contamination of soils and waters, leading to losses of biodiversity (Pinto et al., 2023). There are some natural preservatives like salt, ascorbic acid, and vitamin E, but their range is limited. Therefore, due to the increasing consumer demand for clean-label natural products and the rapid global spread of multidrug-resistant microorganisms including *Escherichia coli*, *Listeria monocytogenes*, *Salmonella* spp., *Staphylococcus aureus*, and *Pseudomonas aeruginosa*, the food industry emphasizes the necessity for natural and environmentally friendly antimicrobial agents (Abrahamian and Goldstein, 2011; Liu et al., 2017).

Effective food preservatives should have a wide range of antimicrobial activities against both gram-negative and gram-positive species. Studies have shown that gram-negative bacteria are generally more resistant to antibacterial agents than gram-positive bacteria due to their distinct cell wall structures (Miller, 2016; Exner et al., 2017). Specifically, gram-negative bacteria possess an additional outer membrane that acts as a protector against harmful substances. Moreover, gram-negative bacteria have porin channels that prevent the entry of toxic chemicals and antibiotics, making them harder to treat (Makridis et al., 2006).

Numerous studies have reported microalgae as a potent source of antimicrobial agents (Schuelter et al., 2019; Dussault et al., 2016; Habibi et al., 2018; Androutsopoulou and Makridis, 2023). For instance, the antibacterial effects of *Tetraselmis* sp. have been observed against *S. aureus* (Kellam and Walker, 1989). Similarly, *Dunaliella salina* has shown antibacterial activity against *Bacillus subtilis*, *S. aureus* and *E. coli* (Ambrico et al., 2020; Herrero et al., 2006). *Spirulina platenis* has been reported to have antimicrobial activity against *S. aureus*, *E. coli*, and *Candida albicans* (Pratita et al., 2019). Moderate to high antimicrobial activity of *Chlorella vulgaris* has also been detected against *B. subtilis*, *S. aureus*, *E. coli*, *P. aeruginosa*, and *C. albicans* (Mashhadinejad et al., 2016).

The antibacterial potential of microalgae results from their bioactive compounds, including proteins (Fadillah et al., 2023), lipids and fatty acids (Amaro et al., 2011; Smith et al., 2010; Bhattacharjee, 2016; Čermák et al., 2015), phycobiliprotein (Najdenski et al., 2013), exopolysaccharides (Najdenski et al., 2013), and phenolic compounds (Vornoli et al., 2023). The latter two are of particular interest to the food industry due to their diverse functional properties, such as texturizing, stabilizing, antioxidant, and antimicrobial effects.

Phenolic compounds are considered among the most appealing natural compounds for use as food preservatives and bioactive ingredients in food and food packaging (Singh et al., 2022; Farvin and Jacobsen, 2013). The diverse bioactivities of phenolic compounds, including antioxidant and antimicrobial effects, attract interest from the food industry for their potential use as high-quality food additives (Nardini, 2022; Shahidi and Dissanayaka, 2023). Moreover, the antibacterial activity of green microalgae *Ettlia pseudoalveolaris* against *E. coli*, *S.* Typhimurium, *S. aureus*, and *E. faecalis* has been mainly attributed to the high content of phenolic compounds (Vornoli et al., 2023).

The precise mechanisms through which phenolic compounds exert antibacterial effects are not fully understood. Nonetheless, these compounds are recognized for targeting diverse cellular sites. It has been proposed that phenolic compounds alter cell membrane permeability or impact intracellular functions by forming hydrogen bonds with enzymes (Lobiuc et al., 2023). Alternatively, they May modify cell wall rigidity, resulting in integrity losses and diverse interactions with the cell membrane. This, in turn, can induce irreversible damage to the cytoplasmic membrane, coagulation of cell content, and even inhibition of intracellular enzymes (Cushnie and Lamb, 2011; Bouarab-Chibane et al., 2019; Lobiuc et al., 2023). Flavonoids can bind to soluble proteins and bacterial cell walls, creating complexes. This results in inhibitory effects on energy metabolism and DNA synthesis, ultimately impacting the synthesis of proteins and RNA (Bouarab-Chibane et al., 2019).

Exopolysaccharides (EPS) are a group of polymeric carbohydrates with major features such as biodegradability, antioxidant and antimicrobial effects, and non-toxicity towards living organisms. These properties give them an advantage in the food industry as biopreservatives (Nešić et al., 2020; Waoo et al., 2023). Recently, polysaccharides have also been investigated as a component of active and intelligent packaging. Their use as primary packaging can potentially replace conventional packaging materials, thus reducing the overall use of synthetic materials (Han, 2014; Nešić et al., 2020).

EPSs are substances that exhibit a wide range of biochemical structures and functions. They come in two primary forms: homopolysaccharides, which are made up of a single repeated monosaccharide, and heteropolysaccharides, composed of two or more distinct sugars. Additionally, they May feature different configurations, such as linearity or branching, and May include various substituents on their backbone, such as methyl or sulfate groups (Delattre et al., 2016). Since they are diverse, they can act through different mechanisms. Despite numerous investigations, the mechanisms underlying the biological effects of EPSs from microalgae remain largely unclear (Laroche, 2022).

EPSs contain different functional groups, such as hydroxyl, phosphate, and carbonyl. These are essential for interactions between microbial EPSs and cell membranes or cell walls of bacterial pathogens, thus contribute to their antimicrobial effects (Riaz Rajoka et al., 2020). For example, antibacterial effects of EPSs can be achieved through the interaction with oligopeptides or acyl-homoserine lactone (quorum sensing signalling molecules) in gram-positive and gram-negative bacteria, respectively (Angelin and Kavitha, 2020). Through this mechanism, EPSs disrupt cell communication and restrain biofilm formation. Thus, microbial EPSs could be effective therapeutic molecules in improving biofilm-related infections (Salimi and Farrokh, 2023). EPSs have a dual function in restraining bacterial pathogens. Firstly, they protect cells from producing a strong host immunological response or act as prebiotics, enhancing the adherence and colonization of beneficial microflora on host cells. This activity prevents the colonization of bacterial pathogens. Secondly, microbial EPSs reduce the autoaggregation of bacterial pathogens, making them more susceptible to host immunological response. EPS-producing probiotics can bind to microbial pathogens, facilitating coaggregation. This, in turn, accelerates antimicrobial functions by obstructing receptors or channels present on the outer membrane of gram-negative pathogenic bacteria (Paynich et al., 2017; Dertli et al., 2015).

Cryptophytes are one of the major primary producers, and play a crucial role in both freshwater and marine food webs (Clay, 2015; Hoef-Emden and Archibald, 2017). They lack strong cell walls of silica or cellulose, making their biomass easily utilizable. Moreover, cryptophyte cells can be easily broken and processed for commercial applications. They contain a wide range of natural bioactive compounds including fatty acids, phycobiliproteins, phenolic compounds, and exopolysaccharides with nutritional value and health-promoting benefits (Peltomaa et al., 2018; Mercier et al., 2022; Abidizadegan et al., 2022; Abidizadegan et al., 2023; Giroldo and Vieira, 2002). Phycobiliproteins, phenolic compounds and exopolysaccharides isolated from cryptophytes have been studied as a promising source of natural antioxidants (Abidizadegan et al., 2022; Abidizadegan et al., 2023). However, from a biotechnological and commercial perspective, research on cryptophytes is still rare, and the potential of cryptophytes requires further comprehensive studies.

To contribute to the ongoing efforts of discovering new natural compounds effective against crucial food pathogens, the main goal of this study was to investigate the antimicrobial effect of total phenolic compounds (TPC) and exopolysaccharides (EPS) isolated from four cryptophyte species.

## 2 Materials and methods

### 2.1 Strains and culturing

Three freshwater cryptophytes from the genus *Cryptomonas*, including *C. ozolinii* (UTEX LB 2782), *C. curvata* (CCAP 979/63) *C*. sp. (*Cryptomonas* sp.; CPCC 336), and a marine cryptophyte *Rhodomonas salina* (CCMP 757) were used in this study. Freshwater strains were grown in a modified MWC medium (MWC: CaCl_2_·2H_2_O, MgSO_4_·7H_2_O, NaHCO_3_, K_2_HPO_4_·3H_2_O, NaNO_3_, Na_2_O_3_Si·5H_2_O, combined trace elements, vitamin mix, buffer TES) (Guillard and Lorenzen, 1972). For the marine strain, F/2 medium [NaNO_3_, NaH_2_PO_4_·2H_2_O, combined trace elements, vitamin mix, sea salt (Dupla Marin Natural Balance, Dohse Aquaristik GmbH & Co. KG, Grafschaft, Germany)] was used (Guillard and Ryther, 1962). The algae were cultivated in 2 L glass bottles in growth cabinets at 20°C under white lights of 100 μmol photons m^−2^ s^−1^, and with gentle bubbling using 2% CO_2_ V/V air. After 10 days, cultures were centrifuged at 4000 × g for 10 min (Heraeus Multifuge 1 S-R, Kendro Laboratory Products, Hamburg, Germany), and the pellets were collected for further experiments.

### 2.2 Extraction method

#### 2.2.1 Phenolic compounds

Microwave-assisted extraction (MAE) was performed as follows in (CEM MARS 6 Microwave Digestion System, Mattehws, North Carolina, United States). Dried algal biomass was loaded in a double-wall vessel, with an appropriate amount of methanol/water (70:30 v/v, ratio of 1 g/50 mL). Extractions were performed at 1200 W, 50°C for 30 min (Gallo et al., 2010; Georgiopoulou et al., 2022; Dang et al., 2017). After MAE, the mixture was centrifuged at 2000 × g for 10 min. Next, the supernatants were used in nitrogen blowdown process (Techne Dri-Block DB100/3 sample concentrator) at 65°C for around 3 h to evaporate the methanol (Sefiane et al., 2003). Finally, the remaining liquids were stored at −20°C and lyophilized (Christ, Beta 2–8 LSCbasic, Ottobeuren, Germany) for 24 h at −60°C and 0.6 mbar to produce phenolic compounds powder.

#### 2.2.2 Exopolysaccharides

For the preparation of EPS, freeze-dried biomass was dissolved in 5 mL of deionized water and shaken for 20 min. Subsequently, the samples underwent centrifugation at 4000 × g for 15 min, and the resulting pellets were re-suspended in 5 mL of a 0.05% NaCl solution. This mixture was then placed on an overhead shaker (New Brunswick Scientific C25KC, Enfield, CT, United States) at a temperature of 60°C for one hour. The samples were then sonicated (Branson 8,510, Brookfield, CT, United States) at 100 W and 20°C for 10 min, after which the suspensions were centrifuged again at 4000 × g for 15 min (Strieth et al., 2020; Chang et al., 2019). Finally, the supernatants obtained were subjected to a lyophilization process, resulting in the EPS powder.

### 2.3 Determination of antimicrobial activity

The pathogenic species used in this study included gram-negative bacteria *Escherichia coli* (HAMBI 862; BM219)*, Salmonella* Typhimurium (HAMBI 224; SH4247), *Pseudomonas fluorescens* (HAMBI 16; CCEB 488), and *Campylobacter jejuni* (HAMBI 2992; E1 3825/1/07); gram-positive bacteria *Staphylococcus aureus* (HAMBI 2319; ATCC 51740) and *Listeria monocytogenes* (HAMBI 2647, ATCC 19112); and fungi *Penicillium roqueforti* (HAMBI 846; FBCC 2516), *Aspergillus versicolor* (HAMBI 3340; FBCC 2548), as well as fungi of the genus *Mucor* (HAMBI 831; FBCC 2504) (Liu et al., 2023; Elbehiry et al., 2023; Moi et al., 2020; Pouris et al., 2024; Punt et al., 2022). The bacterial and fungal species were sourced from the HAMBI Microbial Culture Collection at the University of Helsinki.^1^

Each species was cultured under the following conditions: *E. coli*, *S.* Typhimurium, and *L. monocytogenes* were grown on nutrient agar (VWR Chemicals) plates at 37°C. *C. jejuni* and *S. aureus* were grown on Mueller Hinton Agar (MHA; VWR Chemicals) plates at 37°C. *P. fluorescens* was grown on MHA plate at 30°C. *P. roqueforti*, *A. versicolor*, and *Mucor* sp. were grown on Malt Extract Agar (MEA; VWR Chemicals) plates at 25°C, 27°C, and 25°C, respectively.

To determine MIC by the broth microdilution method, we followed the previously described procedure (Aullybux et al., 2019; Čagalj et al., 2022; Stein et al., 2011; Borman et al., 2017). In brief, bacterial and fungal cultures were prepared and grown at required conditions. After growing the strains, one colony from the cultures was transferred into a sterile saline solution (0.9%), and diluted to a concentration of 1 × 10^6^ CFU/mL. The extracts – total phenolic compounds (TPC) and exopolysaccharides (EPS) – were dissolved in 4% DMSO (VWR Chemicals; in distilled water) and sterile deionized water, respectively.

The procedure involved introducing 50 μL of the appropriate medium (depending on the tested microbial strain: Mueller Hinton broth (MHB; VWR Chemicals) for bacteria and RPMI-1640 (VWR Chemicals) for fungi) into every well of a 96-well microplate. Next, 50 μL of TPC or 50 μL EPS were added to the initial well of each column (in triplicate). A series of 8 two-fold dilutions was prepared by moving 50 μL of the previous dilution to the subsequent well. Then, 50 μL of the prepared inoculum was dispensed into the wells, resulting in a final concentration of 5 × 10^5^ CFU/mL per well. The final concentration of the studied compounds ranged from 1000 to 7.81 μg/mL.

The plates were incubated for 18–20 h for bacterial strains, 48 h for *A. versicolor* and *Mucor* sp., and 72 h for *P. roqueforti* under the specific temperatures required for growth of the pathogenic strains. Non-treated control (50 μL of pathogen inoculum and 50 μL of medium), negative control (50 μL of medium and 50 μL of algal extract), and a blank (100 μL of medium) were prepared in each plate. The final DMSO concentration in the wells varied between 1 and 0.008%. The DMSO test was conducted to confirm that the solvent did not have inhibitory effect on its own. The MIC values of the extracts were determined by the absence of visual turbidity. Each test was performed three times.

The minimum bactericidal concentration (MBC) and the minimum fungicidal concentration (MFC) of the extracts was identified as the lowest concentration at which there was no bacterial/fungal growth. This was observed on agar plates by plating 10 μL bacterial/fungal suspensions from wells where the MIC had been determined and from wells containing higher concentrations of the extract (Garofulić et al., 2021).

#### 2.3.1 Tolerance level

The tolerance level for the tested bacterial strains was determined using the following formula:

TolerancelevelMBCMFC/MIC

## 3 Results

### 3.1 Total phenolic compounds

The MIC and MBC/MFC of TPC extracted from four strains of cryptophyte microalgae against common foodborne pathogens are shown in Table 1. Based on previous studies, MICs <100 μg/mL were categorized as highly active antimicrobial agents, MICs between 101 and 500 μg/mL were moderately active; MICs ranging from 501 to 1,000 μg/mL were labeled as having low activity; and MICs >1,000 μg/mL were classified as inactive (Morales et al., 2008).

**Table 1:** Minimum inhibitory concentration (MIC; μg/ml), minimum bactericidal concentration (MBC; μg/ml), minimum fungicidal concentration (MFC; μg/ml), and tolerance level (TL) of the total phenol compounds (TPC) extracted from four different cryptophyte strains, against representative foodborne pathogens.

Pathogens		*C. ozolinii*			*C. curvata*			*C.* sp.			*R. salina*		
		MIC	MBC	TL	MIC	MBC	TL	MIC	MBC	TL	MIC	MBC	TL
Bacteria													
Gram-negative	*Escherichia coli*	250	250	1	500	500	1	250	250	1	250	500	2
	*Salmonella* Typhimurium	250	250	1	250	250	1	250	500	2	250	250	1
	*Pseudomonas fluorescens*	31.25	125	4	250	250	1	31.25	125	4	125	250	2
	*Campylobacter jejuni*	31.25	31.25	1	31.25	31.25	1	31.25	31.25	1	31.25	62.5	2
Gram-positive	*Staphylococcus aureus*	62.5	250	4	62.5	250	4	62.5	250	4	62.5	250	4
	*Listeria monocytogenes*	31.25	125	4	31.25	125	4	31.25	125	4	31.25	125	4
Fungi		MIC	MFC	TL	MIC	MFC	TL	MIC	MFC	TL	MIC	MFC	TL
*Mucor* sp.		62.5	62.5	1	62.5	125	2	125	125	1	125	125	1
*Penicillium roqueforti*		125	125	1	125	250	2	125	125	1	125	125	1
*Aspergillus versicolor*		62.5	62.5	1	62.5	62.5	1	62.5	125	2	31.25	62.5	2

*Highly active MICs in green, moderate active MICs in yellow.

Gram-negative bacteria *E. coli* and *S.* Typhimurium were the most resistant to total phenolic compounds of algae, as indicated by high MIC values (250–500 μg/mL). *C. jejuni* (the other gram-negative bacterium) was the most susceptible to TPC (MIC 31.25 μg/mL). All four extracts were able to inhibit the growth of fungi, with MIC values of 62.5–125 μg/mL against *Mucor* sp., 125 μg/mL against *P. roqueforti,* and 31.25–62.5 μg/mL against *A. versicolor.*

**Figure 1:**
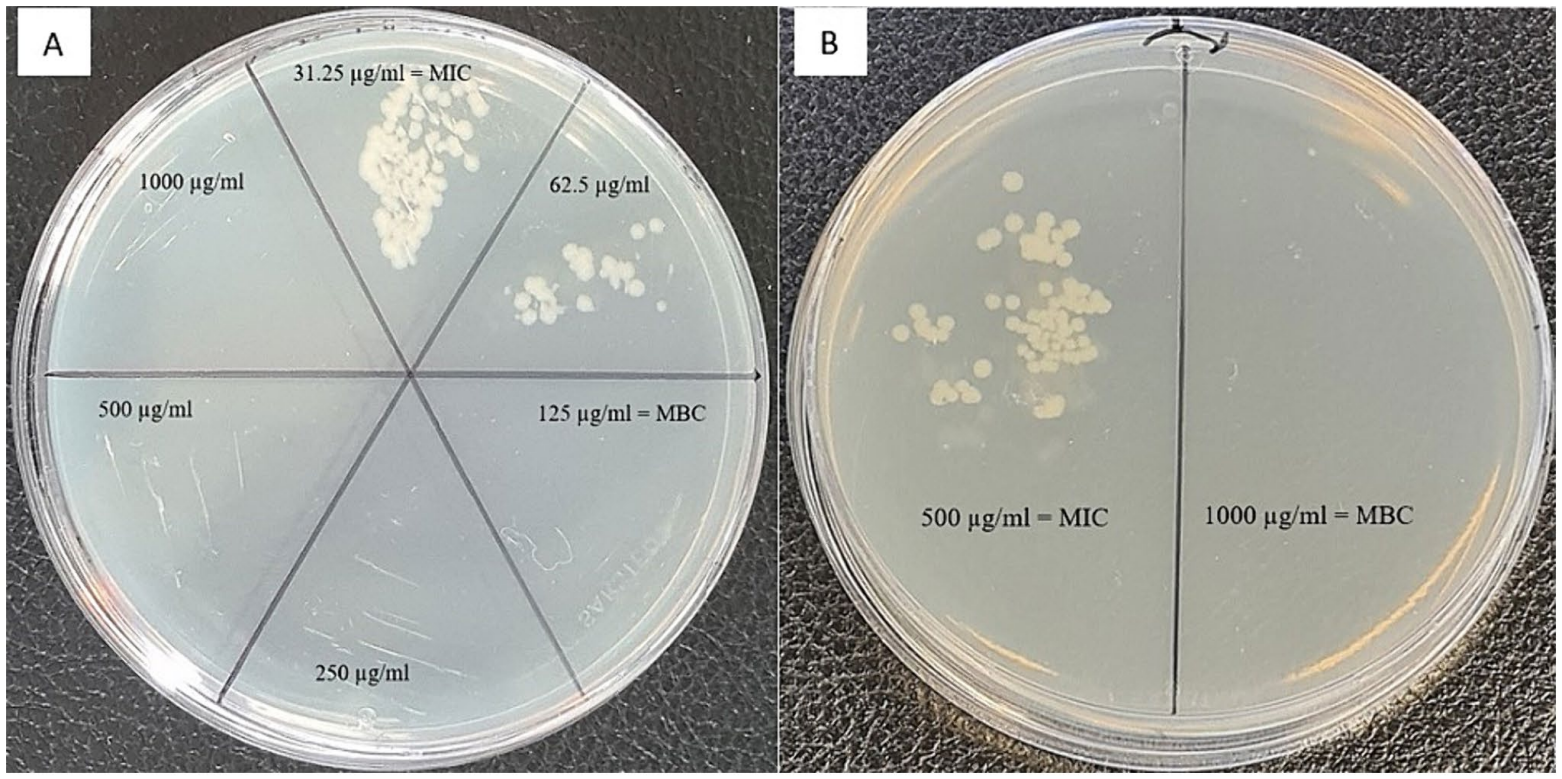
Agar plates displaying the growth of **(A)** the gram-negative bacterium *Pseudomonas fluorescens* after treatment with phenolic compounds extracted from the cryptophyte *Cryptomonas* sp., and **(B)** the gram-negative bacterium *Campylobacter jejuni* after treatment with exopolysaccharides extracted from the cryptophyte *Rhodomonas salina*. Minimum inhibitory concentration (MIC) and minimum bactericidal concentration (MBC) are indicated on the plate.

To further characterize the antibacterial and antifungal properties of the extracts, we assessed minimal bactericidal concentrations. An example of the results for antibacterial activity of TPC extracted from *Cryptomonas* sp. against *P. fluorescens* are shown in Figure 1A Using MIC and MBC values, we calculated tolerance level (TL), which indicates whether test samples have a bactericidal or bacteriostatic effect against the tested strains, as well as a fungicidal or fungistatic effect. When the TL is ≥16, the test agent is considered to have a bacteriostatic or fungistatic effect. Conversely, when TL is ≤4, it signifies bactericidal or fungicidal activity (Mogana et al., 2020; Benjamin et al., 2012). As indicated in Table 1, the tolerance level for all studied samples was less than 4, demonstrating the bactericidal activity of extracted TPC from the studied cryptophytes.

### 3.2 Exopolysaccharides

The MIC and MBC/MFC of EPS extracted from four strains of cryptophyte microalgae against some common foodborne pathogens are presented in Table 2. For bacteria, the MIC and MBC of EPS ranged from 125 to 1,000 μg/mL. The most susceptible strain to EPS extracted from *C. curvata* and *C*. sp. were gram-negative *P. fluorescens* and gram-positive *L. monocytogenes*, with MIC of 125 μg/mL. However, the MIC range for fungi was much wider, from 62.5 to 250 μg/mL, except for *R. salina* against *Mucor* sp. (500–1,000 μg/mL). The most susceptible fungi to EPS was *A. versicolor*, with a MIC value of 62.5 μg/mL against *C. ozolinii*, *C*. sp., and *R. salina* cryptophytes. As evidenced by the data shown in Table 2, the tolerance level for all studied samples is less than 2 (except for *C.* sp. against *L. monocytogenes*, which was ≤4), demonstrating the high bactericidal/fungicidal activity of EPS extracted from the studied cryptophytes. Figure 1B shows an example of an MBC antibacterial activity test for EPS extracted from *R. salina* against *C. jejuni*.

**Table 2:** Minimum inhibitory concentration (MIC; μg/ml), minimum bactericidal concentration (MBC; μg/ml), minimum fungicidal concentration (MFC; μg/ml), and tolerance level (TL) of the exopolysaccharides (EPS) extracted from four different cryptophyte strains against representative foodborne pathogens.

Pathogens		*C. ozolinii*			*C. curvata*			*C.* sp.			*R. salina*		
		MIC	MBC	TL	MIC	MBC	TL	MIC	MBC	TL	MIC	MBC	TL
Bacteria													
Gram-negative	*Escherichia coli*	250	500	2	1,000	1,000	1	500	500	1	1,000	1,000	1
	*Salmonella* Typhimurium	1,000	1,000	1	1,000	1,000	1	1,000	1,000	1	1,000	1,000	1
	*Pseudomonas fluorescens*	250	250	1	125	250	2	1,000	1,000	1	500	1,000	2
	*Campylobacter jejuni*	1,000	1,000	1	500	500	1	1,000	1,000	1	500	1,000	2
Gram-positive	*Staphylococcus aureus*	>1,000	>1,000		250	250	1	>1,000	>1,000		500	500	1
	*Listeria monocytogenes*	250	500	2	500	1,000	2	125	500	4	250	250	1
Fungi		MIC	MFC	TL	MIC	MFC	TL	MIC	MFC	TL	MIC	MFC	TL
*Mucor* sp.		125	125	1	125	125	1	125	250	2	500	1,000	2
*Aspergillus versicolor*		62.5	62.5	1	250	500	2	62.5	125	2	62.5	62.5	1

*Highly active MICs in green, moderate active MICs in yellow, low active MICs in orange, and inactive MICs in red.

## 4 Discussion

To date, very few studies have described the antimicrobial properties of cryptophytes. *Rhodomonas lens* (acetate and hexane extracts) showed antibacterial activity against *L. monocytogenes* and *Enterococcus faecalis* (Fajardo et al., 2020). In this study, the potential of TPC and EPS isolated from four cryptophyte strains – *C. ozolinii*, *C. curvata*, *C*. sp., and *R. salina* – were evaluated against harmful foodborne pathogens. Broth microdilution assay and subculturing broths onto agar plates were used to determine MIC and MBC/MFC. Impressively, almost all TPC and EPS isolated from cryptophytes demonstrated bactericidal activity against both gram-positive and gram-negative bacteria, as well as fungicidal activities against the studied fungi. The only two exceptions were EPS from *C. ozolinii* and *C.* sp., which were shown to be inactive against *S. aureus* at the highest tested concentration of 1,000 μg/mL. Most of the samples were found to be active, with MIC values ranging from 62.5 to 1,000 μg/mL for EPS and 31.25 to 500 μg/mL for TPC. Moreover, TPC and EPS showed high bactericidal/fungicidal activity, with TL ≤4. Studies have shown that gram-negative *Vibrio parahaemolyticus* requires a higher MIC for polyphenols to penetrate the cells compared to the gram-positive *S. aureus* (Wang et al., 2009; Besednova et al., 2020).

The antimicrobial properties of TPC from microalgae are a result of their interaction with proteins, forming complexes that disrupt bacterial cell walls. Additionally, they act as proton exchangers, disturbing the proton motive force and depleting ATP, causing cell death (Rodriguez-Maturino et al., 2015; Rao et al., 2019; Tebou et al., 2017; Chen et al., 2024). They also alter lipid molecules on membranes, enhancing antimicrobial properties by deactivating enzymes and inhibiting ATPase activity, resulting in cell death by disrupting cellular respiration (Pagnussatt et al., 2013; Chen et al., 2024). Furthermore, polyphenolic compounds extracted from microalgae induce the lysis of bacterial cells by disrupting the permeability and integrity of the phospholipid layer in the membrane (Daglia, 2012). This interaction occurs through hydrogen bonding and hydrophobic interactions between the aromatic rings and OH-groups of phloroglucinol units and -NH-groups of bacterial proteins (Venkatesan et al., 2018; Heldt and Piechulla, 2010; Venkatesan et al., 2018).

There is limited documentation of the antibacterial activity of EPS produced by algae, especially cryptophytes. Screening efforts of this study have revealed that exopolysaccharides derived from strains of *C. ozolinii*, *C. curvata*, *C*. sp., and *R. salina* hold potential as effective antimicrobial agents against a range of pathogenic strains. The particularly strong inhibitory effect of EPS on fungi, including *Mucor* sp. and *A. versicolor*, make these cryptophyte EPS a promising avenue for further research.

The potential inhibitory mechanism of EPS can disrupt the structure of the bacterial cell envelope, especially the peptidoglycan layer (Sivasankar et al., 2018). Additionally, interaction of EPS with bacterial cells could block receptors or channels on the outer membrane of the gram-negative bacteria (Medrano et al., 2009). EPS could also potentially hinder cell division, disrupt the cell wall and cytoplasmic membrane, and degrade DNA (Wu et al., 2010).

EPS derived from the cyanobacteria *Gloeocapsa* sp., *Synechocystis* sp., *Nostoc entophytum*, *Nostoc muscorum*, and the red microalga *Rhodella reticulata* have been shown to have inhibitory effects on the growth of *S. aureus*, with MIC values of 125, 1,000, 220, 140, and 250 μg/mL, respectively (Najdenski et al., 2013). Moreover, EPS extracted from the red microalga *Porphyridium marinum* displayed efficiency against *S. aureus* (MIC of 125 μg/mL) (Gargouch et al., 2021). The results of the current study are in line with those of other algae: the inhibitory effect of EPS extracted from *C. curvata* and *R. salina* on *S. aureus* had MIC values of 250 and 500 μg/mL, respectively. Additionally, EPS extracted from the red microalga *Porphyridium marinum* and the cyanobacterium *Gloeocapsa* sp. showed antibacterial effects against *E. coli*, with MIC 1000 and 250 μg/mL (Gargouch et al., 2021; Najdenski et al., 2013). EPS extracted from the studied cryptophytes showed an antibacterial effect against *E. coli*, with MIC of 250, 1,000, 500 and 1,000 μg/mL for *C. ozolinii*, *C. curvata*, *C*. sp., and *R. salina*, respectively. This shows that EPS extracted from cryptophytes have high antibacterial activity against *E. coli*. Furthermore, similar to our results of EPS extracted from *C. ozolinii*, *C. curvata*, *C*. sp., and *R. salina*, EPS from *R. reticulata* demonstrated activity against *S.* Typhimurium, with MIC of 1,000 μg/mL (Najdenski et al., 2013).

There are a few literature reports on antifungal activity of TPC and EPS obtained from microalgae, most of which focus on antifungal effects of crude microalgal extract. It has been reported that the freshwater microalga *Scenedemus obliquus* exhibits significant antifungal effects against various *Aspergillus* species, including *A. flavus*, *A. steynii*, *A. westerdijikiae*, and *A. carbonarius* (Marrez et al., 2019). Furthermore, notable antifungal activities of the green alga *Chlorella vulgaris* were highlighted in *in vitro* studies (Vehapi et al., 2020), while several studies demonstrated the antifungal activity of PCs extracted from *Spirulina* sp. and *Chlorella* sp. against *Aspergillus* species (Danyal et al., 2013; Pagnussatt et al., 2014; Pagnussatt et al., 2016).

The EPS of *Gloeocapsa* sp., *Synechocystis* sp., and *N. entophytum* were highly active against the fungus *Candida albicans*, with MIC of 125, 250, and 220 μg/mL, respectively (Najdenski et al., 2013). This aligns with the antifungal activity of EPS studied in this research against *Mucor* sp. and *A. versicolor*; MIC ranged from 125 to 500 μg/mL and 62.5 to 250 μg/mL, respectively.

The ability of phenolic compounds to inhibit fungal growth depends on their interfere with metabolic pathways, inhibiting amino acid synthesis and impacting protein composition necessary for fungal appressorium development (Pagnussatt et al., 2013). Furthermore, the antifungal activity of EPS is attributed to its interaction with fungi. This interaction affects the respiratory chain and cell division of the fungi, consequently leading to death. Moreover, EPS can hinder the entry of nutrients into pathogenic fungi, thereby slowing down their growth (Liu et al., 2002).

Finally, based on our previous and ongoing research, cryptophytes have demonstrated great potential as candidates to produce TPC and EPS, exhibiting remarkable antioxidant, antibacterial, and antifungal properties. Our findings indicate that *C. pyrenoidifera* and *C.* sp. possess the ability to produce TPC, constituting approximately 30% of their dry weight (DW). Similarly, *C. ozolinii* has been observed to contain TPC at around 25% DW, and both *C. curvata* and *R. salina* have been found to produce EPS accounting for 50% of their DW. Furthermore, the cryptophytes investigated in our study demonstrated significant antioxidant activity in both TPC and EPS, with an IC_50_ value of less than 50 μg/mL (Abidizadegan et al., 2022; Abidizadegan et al., 2023).

## 5 Conclusion

The findings of this study suggest that cryptophyte microalgae, with their total phenolic compounds and exopolysaccharides, have the potential to serve as natural antimicrobial agents (preservatives) in the food industry, including food packaging. These compounds could help in preserving food products and extending their shelf life while offering an eco-friendly alternative to synthetic preservatives. However, further research and comprehensive studies are needed to explore the full potential of cryptophytes and their bioactive compounds in commercial applications.

## Data Availability

The original contributions presented in the study are included in the article/supplementary material, further inquiries can be directed to the corresponding author.

## Footnotes

^1^ https://www.helsinki.fi/en/infrastructures/biodiversity-collections/infrastructures/microbial-domain-biological-resource-centre-hambi

## References

[ref1] Abidizadegan M. Blomster J. Fewer D. Peltomaa E. (2022). Promising biomolecules with high antioxidant capacity derived from cryptophyte algae grown under different light conditions. Biology 11:1112. doi: 10.3390/biology11081112, PMID: 35892969 PMC9331842

[ref2] Abidizadegan M. Blomster J. Peltomaa E. (2023). Effect of micronutrient iron on bioactive compounds isolated from cryptophytes. Front. Plant Sci. 14:1208724. doi: 10.3389/fpls.2023.1208724, PMID: 37575946 PMC10413267

[ref3] Abrahamian F. M. Goldstein E. J. C. (2011). Microbiology of animal bite wound infections. Clin. Microbiol. Rev. 24, 231–246. doi: 10.1128/CMR.00041-10, PMID: 21482724 PMC3122494

[ref4] Amaro H. M. Guedes A. C. Malcata F. X. (2011). “Science against microbial pathogens: communicating current research and technological advances” in Antimicrobial activities of microalgae: An invited review. ed. Méndez-Vilas A. (Badajoz: Forematex Research Center), 1272–1280.

[ref5] Ambrico A. Trupo M. Magarelli R. Balducchi R. Ferraro A. Hristoforou E.. (2020). Effectiveness of *Dunaliella salina* extracts against Bacillus subtilis and bacterial plant pathogens. Pathogens 9:613. doi: 10.3390/pathogens9080613, PMID: 32731345 PMC7459613

[ref6] Androutsopoulou C. Makridis P. (2023). Antibacterial activity against four fish pathogenic bacteria of twelve microalgae species isolated from lagoons in western Greece. Microorganisms 11:1396. doi: 10.3390/microorganisms11061396, PMID: 37374898 PMC10302268

[ref7] Angelin J. Kavitha M. (2020). Exopolysaccharides from probiotic bacteria and their health potential. Int. J. Biol. Macromol. 162, 853–865. doi: 10.1016/j.ijbiomac.2020.06.190, PMID: 32585269 PMC7308007

[ref8] Aullybux A. A. Puchooa D. Bahorun T. Jeewon R. (2019). Phylogenetics and antibacterial properties of exopolysaccharides from marine bacteria isolated from Mauritius seawater. Ann. Microbial. 69, 957–972. doi: 10.1007/s13213-019-01487-2

[ref9] Benjamin T. T. Adebare J. A. Remi Ramota R. Rachael K. (2012). Efficiency of some disinfectants on bacterial wound pathogens. Life Sci J. 9:2012.

[ref10] Besednova N. N. Andryukov B. G. Zaporozhets T. S. Kryzhanovsky S. P. Kuznetsova T. A. Fedyanina L. N.. (2020). Algae polyphenolic compounds and modern antibacterial strategies: current achievements and immediate prospects. Biomedicines 8:342. doi: 10.3390/biomedicines8090342, PMID: 32932759 PMC7554682

[ref11] Bhattacharjee M. (2016). Pharmaceutically valuable bioactive compounds of algae. Asian J. Pharm. Clin. Res. 9, 43–47. doi: 10.22159/ajpcr.2016.v9i6.14507

[ref12] Borman A. M. Fraser M. Palmer M. D. Szekely A. Houldsworth M. Patterson Z.. (2017). MIC distributions and evaluation of fungicidal activity for amphotericin B, itraconazole, voriconazole, posaconazole and caspofungin and 20 species of pathogenic filamentous fungi determined using the CLSI microdilution method. J. Fungi. 3:27. doi: 10.3390/jof3020027, PMID: 29371545 PMC5715917

[ref13] Bouarab-Chibane L. Forquet V. Lantéri P. Clément Y. Léonard-Akkari L. Oulahal N.. (2019). Antibacterial properties of polyphenols: characterization and QSAR (quantitative structure–activity relationship) models. Front. Microbiol. 10:829. doi: 10.3389/fmicb.2019.00829 31057527 PMC6482321

[ref14] Cabral E. M. Oliveira M. Mondala J. R. M. Curtin J. Tiwari B. K. Garcia-Vaquero M. (2021). Antimicrobials from seaweeds for food applications. Mar. Drugs 19:211. doi: 10.3390/md19040211, PMID: 33920329 PMC8070350

[ref15] Čagalj M. Skroza D. Verardo V. Bassi D. Frleta R. Generalić Mekinić I.. (2022). Variations in the composition, antioxidant and antimicrobial activities of *Cystoseira compressa* during seasonal growth. Mar. Drugs 20:64. doi: 10.3390/md20010064, PMID: 35049919 PMC8779577

[ref16] Carocho M. Morales P. Ferreira I. C. (2015). Natural food additives: quo vadis? Trends Food Sci Techno. 45, 284–295. doi: 10.1016/j.tifs.2015.06.007

[ref17] Čermák L. Pražáková Š. Marounek M. Skřivan M. Skřivanová E. (2015). Effect of green alga Planktochlorella nurekis on selected bacteria revealed antibacterial activity in vitro. Czeh J. Anim. Sci. 60, 427–435. doi: 10.17221/8522-CJAS

[ref18] Chang S. P. Sheu H. L. Lee Y. C. (2019). Comparison of EPS extraction efficiencies from *Spirogyra fluviatilis* by chemical and physical extraction methods. Int. J. Biosci. Biochem. Bioinform. 9, 202–209. doi: 10.17706/IJBBB

[ref19] Chen X. Lan W. Xie J. (2024). Natural phenolic compounds: antimicrobial properties, antimicrobial mechanisms, and potential utilization in the preservation of aquatic products. Food Chem. 440:138198. doi: 10.1016/j.foodchem.2023.138198, PMID: 38128429

[ref20] Clay B. L. (2015). “Cryptomonads” in Freshwater algae of North America. eds. Wehr J. D. Sheath R. G. Kociolek P. (Cambridge: Academic Press), 809–850.

[ref21] Cushnie T. P. Lamb A. J. (2011). Recent advances in understanding the antibacterial properties of flavonoids. Int. J. Antimicrob. Agents 38, 99–107. doi: 10.1016/j.ijantimicag.2011.02.014, PMID: 21514796

[ref22] Daglia M. (2012). Polyphenols as antimicrobial agents. Curr. Opin. Biotechnol. 23, 174–181. doi: 10.1016/j.copbio.2011.08.007, PMID: 21925860

[ref23] Dang T. T. Van Vuong Q. Schreider M. J. Bowyer M. C. Van Altena I. A. Scarlett C. J. (2017). Optimization of ultrasound-assisted extraction conditions for phenolic content and antioxidant activities of the alga Hormosira banksii using response surface methodology. J. Appl. Phycol. 29, 3161–3173. doi: 10.1007/s10811-017-1162-y

[ref24] Danyal A. Mubeen U. Malik K. A. (2013). Investigating two native algal species to determine antibiotic susceptibility against some pathogens. Annali di medicina straniera 5, 70–74. doi: 10.19026/crjbs.5.5476

[ref25] Delattre C. Pierre G. Laroche C. Michaud P. (2016). Production, extraction and characterization of microalgal and cyanobacterial exopolysaccharides. Biotechnol. Adv. 34, 1159–1179. doi: 10.1016/j.biotechadv.2016.08.001, PMID: 27530696

[ref26] Dertli E. Mayer M. J. Narbad A. (2015). Impact of the exopolysaccharide layer on biofilms, adhesion and resistance to stress in *Lactobacillus johnsonii* FI9785. BMC Microbiol. 15, 1–9. doi: 10.1186/s12866-015-0347-2 25648083 PMC4326364

[ref27] Dussault D. Vu K. D. Vansach T. Horgen F. D. Lacroix M. (2016). Antimicrobial effects of marine algal extracts and cyanobacterial pure compounds against five foodborne pathogens. Food Chem. 199, 114–118. doi: 10.1016/j.foodchem.2015.11.119, PMID: 26775951

[ref28] Elbehiry A. Abalkhail A. Marzouk E. Elmanssury A. E. Almuzaini A. M. Alfheeaid H.. (2023). An overview of the public health challenges in diagnosing and controlling human foodborne pathogens. Vaccine 11:725. doi: 10.3390/vaccines11040725, PMID: 37112637 PMC10143666

[ref29] Exner M. Bhattacharya S. Christiansen B. Gebel J. Goroncy-Bermes P. Hartemann P.. (2017). Antibiotic resistance: what is so special about multidrug-resistant gram-negative bacteria? GMS Hyg. Infect. Control. 12:Doc05. doi: 10.3205/dgkh000290, PMID: 28451516 PMC5388835

[ref30] Fadillah S. N. Natsir H. Ahmad A. Karim A. Taba P. (2023). Extraction and fractionation of active protein from microalgae Nitzschia sp. as antimicrobial agent. Egypt. J. Chem. 66, 95–100. doi: 10.21608/ejchem.2022.151334.6557

[ref31] Fajardo P. Alonso M. Farabegoli F. Soula M. Ferreira M. Chapela M. (2020). Evaluation of the antimicrobial activity of eight microalga species against aquaculture and food pathogens. Foro dos Recursos Marinos e sa Acuicultura das Rías Galegas. 22, 405–412.

[ref32] Farvin S. K. H. Jacobsen C. (2013). Phenolic compounds and antioxidant activities of selected species of seaweeds from Danish coast. Food Chem. 138, 1670–1681. doi: 10.1016/j.foodchem.2012.10.078, PMID: 23411297

[ref33] Gallo M. Ferracane R. Graziani G. Ritieni A. Fogliano V. (2010). Microwave assisted extraction of phenolic compounds from four different spices. Molecules 15, 6365–6374. doi: 10.3390/molecules15096365, PMID: 20877228 PMC6257672

[ref34] Gargouch N. Elleuch F. Karkouch I. Tabben O. Pichon C. Gardarin C.. (2021). Potential of exopolysaccharide from Porphyridium marinum to contend with bacterial proliferation, biofilm formation, and breast cancer. Mar. Drugs 19:66. doi: 10.3390/md19020066, PMID: 33513982 PMC7911520

[ref35] Garofulić I. E. Malin V. Repajić M. Zorić Z. Pedisić S. Sterniša M.. (2021). Phenolic profile, antioxidant capacity and antimicrobial activity of nettle leaves extracts obtained by advanced extraction techniques. Molecules 26:6153. doi: 10.3390/molecules26206153, PMID: 34684733 PMC8538125

[ref36] Georgiopoulou I. Tzima S. Louli V. Magoulas K. (2022). Process optimization of microwave-assisted extraction of chlorophyll, carotenoid and phenolic compounds from Chlorella vulgaris and comparison with conventional and supercritical fluid extraction. Appl. Sci. 13:2740. doi: 10.3390/app13042740

[ref37] Giroldo D. Vieira A. A. H. (2002). An extracellular sulfated fucose-rich polysaccharide produced by a tropical strain of *Cryptomonas obovata* (Cryptophyceae). J. Appl. Phycol. 14, 185–191. doi: 10.1023/A:1019972109619

[ref38] Guillard R. R. L. Lorenzen C. J. (1972). Yellow-green algae with chlorophyllide c. J. Phycol. 8, 10–14. doi: 10.1111/j.1529-8817.1972.tb03995.x

[ref39] Guillard R. R. L. Ryther J. H. (1962). Studies of marine planktonic diatoms: I. Cyclotella nana Hustedt, and *Detonula confervacea* (Cleve) gran. Can. J. Microbiol. 8, 229–239. doi: 10.1139/m62-029, PMID: 13902807

[ref40] Gupta R. Yadav R. K. (2021). Impact of chemical food preservatives on human health. Palarch’s Archaeol Egypt/Egyptol. 18, 811–818.

[ref41] Habibi Z. Imanpour Namin J. Ramezanpour Z. (2018). Evaluation of antimicrobial activities of microalgae *Scenedesmus dimorphus* extracts against bacterial strains. Caspian Environ. Sci. 16, 25–36.

[ref42] Han J. H. (2014). “Edible films and coatings” in Innovations in food packaging. ed. Han J. H. (Amsterdam: Academic Press), 213–255.

[ref43] Heldt H. W. Piechulla B. (2010). Plant biochemistry. San Diego: Academic Press.

[ref44] Herrero M. Ibáñez E. Cifuentes A. Reglero G. Santoyo S. (2006). *Dunaliella salina* microalga pressurized liquid extracts as potential antimicrobials. Food Prot. 69, 2471–2477. doi: 10.4315/0362-028x-69.10.2471 17066930

[ref45] Hoef-Emden K. Archibald J. M. (2017). “Cryptophyta (Cryptomonads)” in Handbook of the Protists. eds. Archibald J. Simpson A. Slamovits C. (Cham: springer), 851–891.

[ref46] Kellam S. J. Walker J. M. (1989). Antibacterial activity from marine microalgae in laboratory culture. Br. Phycol. J. 24, 191–194. doi: 10.1080/00071618900650181, PMID: 33513982

[ref47] Laroche C. (2022). Exopolysaccharides from microalgae and cyanobacteria: diversity of strains, production strategies, and applications. Mar. Drugs 20:336. doi: 10.3390/md20050336, PMID: 35621987 PMC9148076

[ref48] Liu Q. Meng X. Li Y. Zhao N. Tang Y. Li B. (2017). Antibacterial and antifungal activities of spices. Int. J. Mol. Sci. 18:1283. doi: 10.3390/ijms18061283, PMID: 28621716 PMC5486105

[ref49] Liu A. Xu R. Zhang S. Wang Y. Hu B. Ao X.. (2002). Antifungal mechanisms and application of lactic acid bacteria in bakery products: a review. Front. Microbiol. 13:924398. doi: 10.3389/fmicb.2022.924398 PMC9244174 35783382

[ref50] Liu X. Yao H. Zhao X. Ge C. (2023). Biofilm formation and control of foodborne pathogenic bacteria. Molecules 28:2432. doi: 10.3390/molecules28062432, PMID: 36985403 PMC10058477

[ref51] Lobiuc A. Pavăl N. E. Mangalagiu I. I. Gheorghiță R. Teliban G. C. Amăriucăi-Mantu D.. (2023). Future antimicrobials: natural and functionalized phenolics. Molecules 28:1114. doi: 10.3390/molecules28031114, PMID: 36770780 PMC9920704

[ref52] Makridis P. Costa R. A. Dinis M. T. (2006). Microbial conditions and antimicrobial activity in cultures of two microalgae species, Tetraselmis chuii and Chlorella minutissima, and effect on bacterial load of enriched Artemia metanauplii. Aquaculture 255, 76–81. doi: 10.1016/j.aquaculture.2005.12.010

[ref53] Marrez D. A. Naguib M. M. Sultan Y. Y. Higazy A. M. (2019). Antimicrobial and anticancer activities of *Scenedesmus obliquus* metabolites. Heliyon 5:e01404. doi: 10.1016/j.heliyon.2019.e01404, PMID: 30976685 PMC6441795

[ref54] Martins N. Roriz C. L. Morales P. Barros L. Ferreira I. C. F. R. (2017). Coloring attributes of betalains: a key emphasis on stability and future applications. Food Funct. 8, 1357–1372. doi: 10.1039/C7FO00144D, PMID: 28262892

[ref55] Mashhadinejad A. Zamani H. Sarmad J. (2016). Effect of growth conditions and extraction solvents on enhancement of antimicrobial activity of the microalgae *Chlorella vulgaris*. Pharm Biomed Res. 2, 65–73. doi: 10.18869/acadpub.pbr.2.4.65

[ref56] Medrano M. Hamet M. F. Abraham A. G. Pérez P. F. (2009). Kefiran protects Caco-2 cells from cytopathic effects induced by *Bacillus cereus* infection. Antonie Van Leeuwenhoek 96, 505–513. doi: 10.1007/s10482-009-9366-z, PMID: 19633916

[ref57] Mercier L. Peltomaa E. Ojala A. (2022). Comparative analysis of phycoerythrin production in cryptophytes. J. Appl. Phycol. 34, 789–797. doi: 10.1007/s10811-021-02657-z, PMID: 31845015

[ref58] Miller S. I. (2016). Antibiotic resistance and regulation of the gram-negative bacterial outer membrane barrier by host innate immune molecules. MBio 7:e01541. doi: 10.1128/mBio.01541-16, PMID: 27677793 PMC5040116

[ref59] Mogana R. Adhikari A. Tzar M. N. Ramliza R. Wiart C. (2020). Antibacterial activities of the extracts, fractions and isolated compounds from Canarium patentinervium Miq. Against bacterial clinical isolates. BMC Complement Med. Ther. 20:5. doi: 10.1186/s12906-020-2837-5 32059725 PMC7076860

[ref60] Moi I. M. Ibrahim Z. Abubakar B. M. Katagum Y. M. Abdullahi A. Yiga G. A.. (2020). Properties of foodborne pathogens and their diseases. IntechOpen. doi: 10.5772/intechopen.105694

[ref61] Morales G. Paredes A. Sierra P. Loyola L. A. (2008). Antimicrobial activity of three baccharis species used in the traditional medicine of northern Chile. Molecules 13, 790–794. doi: 10.3390/molecules13040790, PMID: 18463580 PMC6245405

[ref62] Najdenski H. M. Gigova L. G. Iliev I. I. Pilarski P. S. Lukavský J. Tsvetkova I. V.. (2013). Antibacterial and antifungal activities of selected microalgae and cyanobacteria. Int. J. Food Sci. Tech. 48, 1533–1540. doi: 10.1111/ijfs.12122, PMID: 26902670

[ref63] Nardini M. (2022). Phenolic compounds in food: characterization and health benefits. Molecules 27:783. doi: 10.3390/molecules27030783, PMID: 35164044 PMC8839921

[ref64] Nešić A. Cabrera-Barjas G. Dimitrijević-Branković S. Davidović S. Radovanović N. Delattre C. (2020). Prospect of polysaccharide-based materials as advanced food packaging. Molecules 25:135. doi: 10.3390/molecules25010135 PMC6983128 31905753

[ref65] Pagnussatt F. A. de Lima V. R. Dora C. L. Costa J. A. Putaux J. L. Badiale-Furlong E. (2016). Assessment of the encapsulation effect of phenolic compounds from Spirulina sp. LEB-18 on their antifusarium activities. Food Chem. 211, 616–623. doi: 10.1016/j.foodchem.2016.05.098, PMID: 27283675

[ref66] Pagnussatt F. A. Del Ponte E. M. Garda-Buffon J. Badiale-Furlong E. (2014). Inhibition of Fusarium graminearum growth and mycotoxin production by phenolic extract from Spirulina sp. Pestic. Biochem. Phys. 108, 21–26. doi: 10.1016/j.pestbp.2013.11.002, PMID: 24485311

[ref67] Pagnussatt F. A. Kupski L. Darley F. T. Filoda P. F. Ponte E. M. D. Garda-Buffon J.. (2013). Fusarium graminearum growth inhibition mechanism using phenolic compounds from Spirulina sp. Cienc. Tecnol. Aliment. 33, 75–80. doi: 10.1590/S0101-20612013000500012, PMID: 27283675

[ref68] Paynich M. L. Jones-Burrage S. E. Knight K. L. (2017). Exopolysaccharide from *Bacillus subtilis* induces anti-inflammatory M2 macrophages that prevent T cell–mediated disease. J Immun. 198, 2689–2698. doi: 10.4049/jimmunol.1601641, PMID: 28202619 PMC5360499

[ref69] Peltomaa E. Johnson M. D. Taipale S. J. (2018). Marine cryptophytes are great sources of EPA and DHA. Mar. Drugs 16:3. doi: 10.3390/md16010003 PMC5793051 29278384

[ref70] Pinto L. Tapia-Rodríguez M. R. Baruzzi F. Ayala-Zavala J. F. (2023). Plant antimicrobials for food quality and safety: recent views and future challenges. Food Secur. 12:2315. doi: 10.3390/foods12122315, PMID: 37372527 PMC10297530

[ref71] Pouris J. Kolyva F. Bratahou S. Vogiatzi C. A. Chaniotis D. Beloukas A. (2024). The role of fungi in food production and processing. Appl. Sci. 14:5046. doi: 10.3390/app14125046, PMID: 39254357

[ref72] Pratita A. T. K. Lestari Y. Setiawati M. R. Widyaningrum P. (2019). Potential of autotroph microalgae (Spirulina plantentis) as antimicrobial agent. Phys Conf Ser. 1185:012173. doi: 10.1088/1742-6596/1185/1/012173

[ref73] Punt M. Seekles S. J. van Dam J. L. de Adelhart Toorop C. Martina R. R. Houbraken J.. (2022). High sorbic acid resistance of Pencillium roqueforti is mediated by the SORBUS gene cluster. PLoS Genet. 18:e1010086. doi: 10.1371/journal.pgen.1010086, PMID: 35704633 PMC9200314

[ref74] Rao J. Chen B. McClement D. J. (2019). Improving the efficacy of essential oils as antimicrobials in food: mechanism of action. Annu. Rev. Food Sci. Technol. 10, 365–387. doi: 10.1146/annurev-food-032818-121727, PMID: 30653350

[ref75] Riaz Rajoka M. S. Mehwish H. M. Zhang H. Ashraf M. Fang H. Zeng X.. (2020). Antibacterial and antioxidant activity of exopolysaccharide mediated silver nanoparticle synthesized by *Lactobacillus brevis* isolated from Chinese koumiss. Colloids Surf. B Biointerfaces 186:110734. doi: 10.1016/j.colsurfb.2019.110734, PMID: 31865119

[ref76] Rodriguez-Maturino A. Troncoso-Rojas R. Sánchez-Estrada A. González-Mendoza D. Ruiz-Sanchez E. Zamora-Bustillos R.. (2015). Antifungal effect of phenolic and carotenoids extracts from chiltepin (Capsicum annum var. glabriusculum) on Alternaria alternata and Fusarium oxysporum. Rev. Argent. Microbiol. 47, 72–77. doi: 10.1016/j.ram.2014.12.005, PMID: 25705046

[ref77] Salimi F. Farrokh P. (2023). Recent advances in the biological activities of microbial exopolysaccharides. World J. Microbiol. Biotechnol. 39:213. doi: 10.1007/s11274-023-03660-x 37256348 PMC10230149

[ref78] Sambu S. Hemaram U. Murugan R. Alsofi A. A. (2022). Toxicological and teratogenic effect of various food additives: an updated review. Biomed. Res. Int. 2022:6829409. doi: 10.1155/2022/6829409, PMID: 35782077 PMC9249520

[ref79] Schuelter A. R. Kroumov A. D. Hinterholz C. L. Fiorini A. Trigueros D. E. G. Vendruscolo E. G.. (2019). Isolation and identification of new microalgae strains with antibacterial activity on food-borne pathogens. Engineering approach to optimize synthesis of desired metabolites. Biochem Eng. 144, 28–39. doi: 10.1016/j.bej.2019.01.007

[ref80] Sefiane K. Tadrist L. Douglas M. (2003). Experimental study of evaporating water-ethanol mixture sessile drop: influence of concentration. Int. J. Heat Mass Transf. 46, 4527–4534. doi: 10.1016/S0017-9310(03)00267-9

[ref81] Shahidi F. Dissanayaka C. S. (2023). Phenolic-protein interactions: insight from in-silico analyses–a review. Food Prod. Process. Nutr. 2. doi: 10.1186/s43014-022-00121-0

[ref82] Singh A. K. Kim J. Y. Lee Y. S. (2022). Phenolic compounds in active packaging and edible films/coatings: natural bioactive molecules and novel packaging ingredients. Molecules 27:7513. doi: 10.3390/molecules27217513, PMID: 36364340 PMC9655785

[ref83] Sivasankar P. Seedevi P. Poongodi S. Sivakumar M. Murugan T. Sivakumar L.. (2018). Characterization, antimicrobial and antioxidant property of exopolysaccharide mediated silver nanoparticles synthesized by *Streptomyces violaceus* MM72. Carbohydr. Polym. 181, 752–759. doi: 10.1016/j.carbpol.2017.11.082, PMID: 29254032

[ref84] Smith V. J. Desbois A. P. Dyrynda E. A. (2010). Conventional and unconventional antimicrobials from fish, marine invertebrates and micro-algae. Mar. Drugs 8, 1213–1262. doi: 10.3390/md8041213, PMID: 20479976 PMC2866484

[ref85] Stein E. M. Colepicolo P. Afonso F. A. K. Fujii M. T. (2011). Screening for antifungal activities of extracts of the Brazilian seaweed genus Laurencia (Ceramiales, Rhodophyta). Rev. Bras 21, 290–295. doi: 10.1590/S0102-695X2011005000085

[ref86] Strieth D. Stiefelmaier J. Wrabl B. Schwing J. Schmeckebier A. Nonno S. D.. (2020). A new strategy for a combined isolation of EPS and pigments from cyanobacteria. J. Appl. Phycol. 32, 1729–1740. doi: 10.1007/s10811-020-02063-x

[ref87] Taylor T. M. Ravishankar S. Bhargava K. Juneja V. K. (2019). “Chemical preservatives and natural food antimicrobials” in Food microbiology: Fundamentals and Frontiers. eds. Doyle M. P. Diez-Gonzalez F. Hill C. (Washington DC: ASM Press), 705–731.

[ref88] Tebou P. Tamokou J. Ngnokam D. Voutquenne-Nazabadioko L. Kuiate J. Bag P. (2017). Flavonoids from Maytenus buchananii as potential cholera chemotherapeutic agents. S. Afr. J. Bot. 109, 58–65. doi: 10.1016/j.sajb.2016.12.019

[ref89] Vehapi M. Koçer A. T. Yılmaz A. Özçimen D. (2020). Investigation of the antifungal effects of algal extracts on apple-infecting fungi. Arch. Microbiol. 202, 455–471. doi: 10.1007/s00203-019-01760-7, PMID: 31696248

[ref90] Venkatesan J. Keekan K. K. Anil S. Bhatnagar I. Kim S. K. (2018). Phlorotannins. Encycl. Food Chem. 2019, 515–527. doi: 10.1016/B978-0-08-100596-5.22360-3

[ref91] Vornoli A. Grande T. Lubrano V. Vizzarri F. Gorelli C. Raffaelli A.. (2023). In vitro characterization of antioxidant, antibacterial and antimutagenic activities of the green microalga Ettlia pseudoalveolaris. Antioxidants 12:1308. doi: 10.3390/antiox12061308, PMID: 37372038 PMC10294935

[ref92] Wang Y. Xu Z. Bach S. J. McAllister T. A. (2009). Sensitivity of *Escherichia coli* to seaweed (*Ascophyllum nodosum*) phlorotannins and terrestrial tannins. Asian-Aust J Anim Sci. 22, 238–245. doi: 10.5713/ajas.2009.80213

[ref93] Waoo A. A. Singh S. Pandey A. Kant G. Choure K. Amesho K. T.. (2023). Microbial exopolysaccharides in the biomedical and pharmaceutical industries. Heliyon 9:e18613. doi: 10.1016/j.heliyon.2023.e18613, PMID: 37593641 PMC10432183

[ref94] Wu M. H. Pan T. M. Wu Y. J. Chang S. J. Chang M. S. Hu C. Y. (2010). Exopolysaccharide activities from probiotic bifidobacterium: immunomodulatory effects (on J774A.1 macrophages) and antimicrobial properties. Int. J. Food Microbiol. 144, 104–110. doi: 10.1016/j.ijfoodmicro.2010.09.003, PMID: 20884069

